# The impact of holistic review on correlations between doctoral student outcomes, and GPA and GRE scores in the biomedical sciences

**DOI:** 10.1371/journal.pone.0279258

**Published:** 2022-12-16

**Authors:** Taylor Walters, Antonio Abeyta, Andrew J. Bean, Marenda A. Wilson

**Affiliations:** 1 College of Arts and Sciences, Oberlin College and Conservatory, Oberlin, Ohio, United States of America; 2 The Graduate College, Rush University, Chicago, Illinois, United States of America; 3 Deans’ Office, The University of Texas MD Anderson Cancer Center UTHealth Graduate School of Biomedical Sciences, Houston, Texas, United States of America; 4 Department of Neurobiology and Anatomy, Programs in Neuroscience, Cell Biology and Biochemistry, McGovern Medical School at The University of Texas Health Science Center at Houston, Houston, Texas, United States of America; 5 Department of Pediatrics, The University of Texas MD Anderson Cancer Center, Houston, Texas, United States of America; Ege University Faculty of Medicine, TURKEY

## Abstract

Graduate admissions committees throughout the United States examine both quantitative and qualitative data from applicants to make admissions determinations. A number of recent studies have examined the ability of commonly used quantitative metrics such as the GRE and undergraduate GPA to predict the likelihood of applicant success in graduate programs. We examined whether an admissions committee could predict applicant success at The University of Texas MD Anderson Cancer Center UTHealth Graduate School of Biomedical Sciences based on quantitative metrics. We analyzed the predictive validity of admissions scores, undergraduate GPA, and the GRE for student success. We observed nuanced differences based on gender, ethnicity, race, and citizenship status. The scores assigned to applicants by the admissions committee could not predict time to degree in PhD students regardless of demographic group. Undergraduate GPA was correlated with time to degree in some instances. Interestingly, while GRE scores could predict time to degree, GRE percentile scores could predict both time to degree and PhD candidacy examination results. These findings suggest that there is a level of nuance that is required for interpretation of these quantitative metrics by admissions committees.

## Introduction

The Graduate Record Examination (GRE) is a commonly used standardized test that has often been used as part of the information considered in the decision-making process for graduate admissions in programs focused on science, technology, engineering, and mathematics (STEM). The GRE measures verbal reasoning, quantitative reasoning, and analytical writing. The Verbal Reasoning section examines the ability of an applicant to comprehend complex literature and form conclusions based on the information provided, which are important skills for students in programs that require critical thinking and critical analysis. The Quantitative Reasoning section examines the ability to analyze data and perform mathematic functions, which are important skills for organizing and analyzing data sets that are a cornerstone of graduate education. The Analytical Writing section assesses an applicant’s ability to think critically about questions and articulate their thoughts in writing, skills that are important for conducting research and communicating with specialized and lay audiences.

The Educational Testing Service (ETS) provides students with a GRE score and percentile for each section. While the GRE score is simply a numerical measure of how many questions the student answered correctly (currently scaled from 130–170 points per section), the GRE percentile compares the student’s performance on the GRE to that of other students over a span of three years [1]. While the meaning of a given GRE score could change for an admissions committee depending on the scores of other students, the significance of the percentile in which a student scores remains consistent over a period of time. However, some Programs emphasize GRE scores in their admissions criteria (e.g. Wake Forest University [2]; Northeastern University [3]; Duke University [4]; University of Notre Dame [5]; Purdue University [6]), while others use GRE percentiles for consideration of admission (University of Cincinnati [7]; University of South Carolina [8]).

Graduate school admissions committees are faced with hundreds to thousands of applications each year and must decide which students will be the most likely to succeed in programs at their institutions based on an application packet. The packet typically includes the students’ personal information, academic transcripts, standardized test scores, curricula vitae, letters of recommendation, and essays that explain why the student wants to attend graduate school, and/or a research statement. Admissions committees must then select the “best” applicants based on this information. In 2016, the annual applicant pool of doctoral graduate programs was ~2.2 million [9] for approximately 5,000 doctoral programs [10]. The admissions process, including applicant review, can take place over the span of about six months each year and 58% of admissions committees report time as the rate-limiting factor in applicant review [11]. The difficulty of reviewing a large group of applicants in a short period of time, combined with the volunteer faculty workforce that comprises many admissions committees, can result in committees implementing cutoff scores on quantitative metrics to reduce the size of the applicant pool [12].

Quantitative metrics meant to assess aspects of academic performance can be biased. There is evidence of bias in undergraduate GPA based on socioeconomic status [13] and first-generation student status [14], in addition to gender and racial bias in the GRE [15]. On average, women and underrepresented minorities (URMs) score lower on the GRE than well-represented male students [15]. It is therefore possible that the underrepresentation of certain demographic groups in STEM fields is due, in part, to bias in the GRE [12, 16, 17]. The importance placed on GRE scores and percentiles by graduate admissions committees may therefore have contributed to the underrepresentation of certain demographic groups in STEM [12, 16, 17]. The lack of URMs in the STEM workforce could therefore be explained, in part, by the underrepresentation of URMs in graduate programs. While enrollment of URMs in graduate programs in the biological sciences has steadily increased since 2000, URMs make up 30% of the United States’ population but only 8.5% of doctoral students, 4% of postdoctoral fellows, 5% of principal investigators on research grants, and 13% of the STEM workforce [18–23]. The use of cutoff scores in the admissions process, combined with the reported cultural bias of the GRE, could significantly impact the representation of underrepresented groups in STEM fields. In this regard, a metrics-based review of applicants with quantitative cutoffs excluded two times the number of URM students’ applications from being reviewed when compared to well-represented students at The University of Texas MD Anderson Cancer Center UTHealth Graduate School of Biomedical Sciences (GSBS) [24].

We recently tracked a statistically significant increase in diversity in the doctoral program at the GSBS resulting from multiple initiatives that overhauled the admissions process [25]. Significant increases in the number of URM students were offered interviews following the change from a metrics-based admissions process that used GRE score cutoffs, to a holistic review that used GRE results as one factor among many others in a review for admission. A whole-file review enables consideration of the entire application including personal statements, letters of recommendation, evidence of research participation, productivity, and traditional quantitative metrics like grade point average (GPA) and GRE scores. This increases the emphasis on skills and experiences that are thought to be relevant to success in graduate school while decreasing the reliance on a few quantitative metrics [24]. A similar result has been recorded at other programs that have implemented a holistic review [26, 27]. In our previous work, we provided a model for holistic review that mitigates the reported cultural bias of the GRE without increasing the workload for an admissions committee [24]. We found that a well-informed admissions committee with a reasonable workload was able to assess applicants in a manner that correlated with GRE scores but could still mitigate the GRE score bias that has been reported.

A number of recent studies have questioned the predictive validity of the GRE [28–32]. While it is difficult to quantify success in graduate level programs, GRE scores have not proven useful in predicting number of publications, time to defense, or PhD candidacy exam results [28–30]. However, GRE scores are correlated with higher scores in first year graduate courses and on exit exams [31, 32]. Because of the importance that quantitative metrics have played in the graduate school admissions process, it is essential for admissions committees to understand the extent to which these metrics can predict outcomes. While many studies have reported that these metrics are inaccurate predictors of graduate success [28–30], we wanted to understand if the predictive validity of quantitative metrics changed when used in the context of a holistic review that mitigates biases.

To analyze the effectiveness of using quantitative measures such as the GRE and GPA in graduate school admissions, we examined data over a six-year period (2007–2012) at the University of Texas MD Anderson Cancer Center UTHealth GSBS for which we possess both entry and outcome information for students. We wanted to determine whether the GRE and GPA could predict graduate school outcomes. We present data that show: 1) use of quantitative metrics in graduate school admissions requires careful consideration and a nuanced approach as they can be predictive of graduate school success in non-obvious ways and 2) GRE scores and GRE percentiles are differently correlated with outcomes depending on the population being examined.

## Methods

### The graduate school

The University of Texas MD Anderson Cancer Center UTHealth Graduate School of Biomedical Sciences (GSBS) is the degree-granting entity of The University of Texas MD Anderson Cancer Center and The University of Texas Health Science Center at Houston. The GSBS offers three master’s programs, a Medical Physics PhD Program, and eight biomedical sciences PhD Programs in Biochemistry and Cell Biology, Cancer Biology, Genetics and Epigenetics, Immunology, Microbiology and Infectious Diseases, Neuroscience, Quantitative Sciences, and Therapeutics and Pharmacology. The graduate school has a centralized biomedical sciences admissions process in which students that are admitted to the graduate school can join any of the biomedical sciences programs.

### Data sources

The data presented were extracted from the Admissions and Student Databases as previously described [25]. Briefly, custom database systems are managed at the graduate school on the Microsoft Office Access platform. Each individual is assigned a unique identifier and sub identifiers are used to distinguish between applicant, student, and alumni information.

### Definitions

*URM student/applicant*: An American citizen who identified as Black/African American, Native

(American Indian, Alaskan Native, Hawaiian Native), Pacific Islander, or Hispanic in the application for admission.

*Well-represented Student/applicant*: An American citizen who identified as white (non-Hispanic) or Asian American in the application for admission.

*International student/applicant*: An applicant who is not an American citizen or permanent resident. Neither racial nor ethnic data are collected from international applicants in the application for admission. As such, international students are not considered in analyses of racial and ethnic students that are considered under-represented in the biomedical sciences in the United States, but as a standalone group.

*Domestic student/applicant*: An applicant who is an American citizen or permanent resident. Racial and ethnic data are collected voluntarily in the application for admission.

### Participants

Verbal and Quantitative Reasoning GRE scores and percentiles were collected by querying the student database for the appropriate information. Any student records that were missing data such as GRE scores or grade point average were removed from the study before the data were analyzed (S1 Fig.).

The GRE Scores of entering doctoral students from 2007–2012 were collected and analyzed. A total of 528 student records were reviewed. Ninety-six records were removed from the data because of a lack of GRE scores or admissions committee scores as follows: Thirty-nine of these records belonged to MD/PhD applicants who were not required to take the GRE to be reviewed for admission; fifty-seven records were removed because they did not have an admissions committee score in the database. In addition, thirty-nine admissions scores were identified as outliers by statistical analysis software and removed for a final data set of 286 records (see **Outliers** below). After 2011, the GRE’s scoring system was changed from a scale of 200–800 points per section to 130–170 points per section. As a result, six additional records were removed because their scores were representative of the new scoring system and therefore were not able to be compared to the older scores based on raw score. One hundred and one records of students that did not complete the PhD Program, were currently enrolled, or left the program with a MS degree were then removed so that our analyses only considered students that graduated with a doctoral degree.

This is a retrospective study of anonymized archived data from the The University of Texas MD Anderson Cancer Center UTHealth Graduate School of Biomedical Sciences that did not require IRB approval because the protocols for data collection did not contain hypothesis-based research.

### Outliers

As outlined in our previous study [24], we used the automated ROUT method included in the PRISM software to test the data for the presence of outliers of admissions committee scores, which could skew our data. The false discovery rate for outlier detection (Q) was set to 1%. After removing the 96 students without a GRE score, 432 students were reviewed for the presence of outliers. ROUT detected 39 outliers that were removed before statistical analysis was performed.

### Sample

See detailed description in the *Participants* section. Linear regression analysis was used to examine potential trends between GRE scores, GRE percentiles, normalized admissions scores or GPA and outcomes between selected student groups. The D’Agostino & Pearson omnibus and Shapiro-Wilk normality tests were used to test for normality regarding outcomes in the sample. The Pearson correlation coefficient was calculated to determine the relationship between GRE scores, GRE percentiles, admissions scores or GPA (undergraduate and graduate) and time to degree. Candidacy exam results were divided into students who either passed or failed the exam. A Mann-Whitney test was then used to test for statistically significant differences between mean GRE scores, percentiles, and undergraduate GPA and candidacy exam results. Other variables were also observed such as gender, race, ethnicity, and citizenship status within the samples.

#### Predictive metrics

The input variables used in this study were GPA and scores and percentiles of applicants on both the Quantitative and Verbal Reasoning GRE sections. GRE scores and percentiles were examined to normalize variances that could occur between tests.

#### Performance metrics

The output variables used in the statistical analyses of each data set were either the amount of time it took for each student to earn their doctoral degree, or the student’s candidacy examination result.

### Statistical analyses

Statistical analyses were performed using the PRISM software v. 8.43 (GraphPad Software, La Jolla, CA) as described in the *Sample* section. Briefly, extracted data was exported to Microsoft Excel and statistical analyses were there conducted using PRISM (D’Agostino & Pearson omnibus and Shapiro-Wilk normality, Pearson correlation coefficient, Mann-Whitney). Significance was assigned to *p* values ≤ 0.05.

## Results

### An admissions committee can mitigate GRE score variances between demographic groups, but admissions committee scores alone do not predict PhD student outcomes

**Fig 1:**
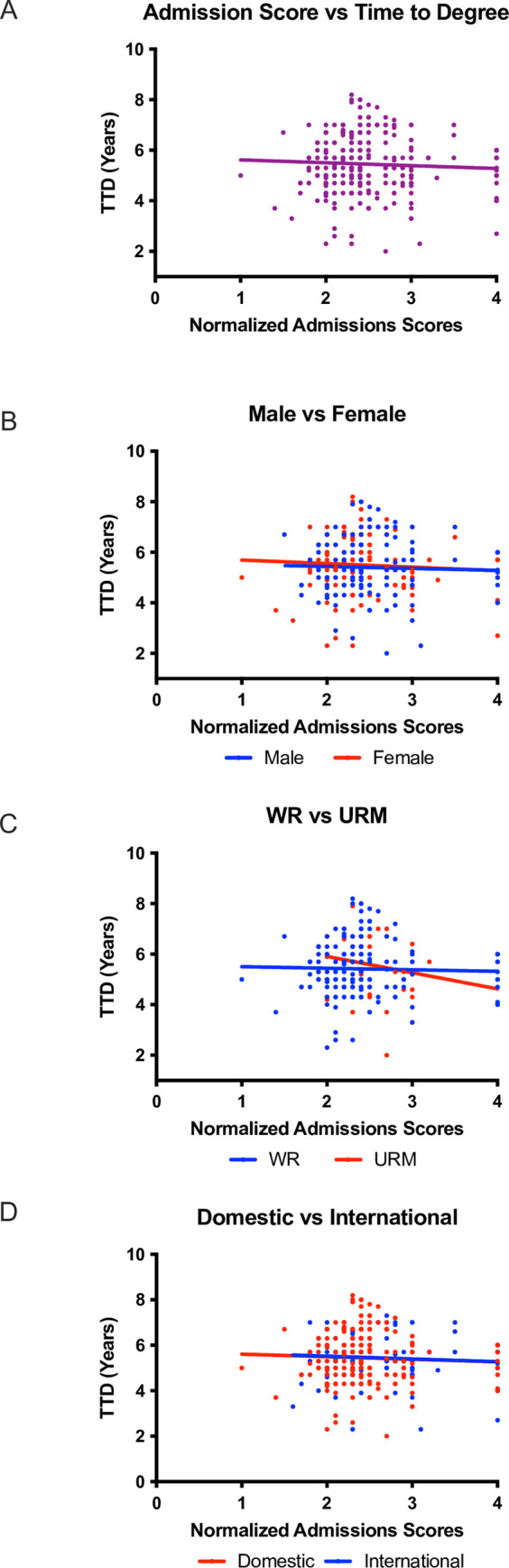
Normalized admissions scores from application review are not correlated to the time it takes to earn a doctoral degree. Time to degree is plotted as a function of admissions scores (A). The mean time to degree was 5.447 years with a standard deviation of 1.126. The relationship between admissions scores and time to degree was further plotted based on applicant gender (B), representation (C), and citizenship (D).

In our previous work, we reviewed and assessed holistic applicant review by the admissions committee at the University of Texas MD Anderson Cancer Center Graduate School of Biomedical Sciences [24, 25]. However, we wanted to further analyze the data to determine whether an admissions committee could predict graduate school outcomes by analyzing the predictive validity of the scores assigned to applicants by the committee. Admissions scores (x-axis) were used as the input variable as they are a normalized score that represents all aspects of the application such as GRE performance, GPA, and letters of recommendation. Time to degree (TTD; y-axis) was used as an indication of graduate school success as it has been used in multiple studies as a quantitative graduate school outcome [28–30, 33]. We used a scatterplot analysis to assess this relationship (Fig 1). To visualize trends between admissions scores and TTD, linear regression analyses were added to the scatterplots. A positively sloped trend line would suggest that the lower (more favorable) a student’s admissions committee score, the less time it would take for the student to complete the PhD program. A horizontal trend line would suggest that there is no relationship between admissions committee scores and TTD. A negatively sloped trend line would suggest that the higher (less favorable) the student’s admissions committee score, the earlier the student would graduate with a PhD. We observed no significant correlation between admissions committee scores and TTD among all students in our analysis (Fig 1A). There was also no significant correlation when analyzed by gender (Fig 1B), race/ethnicity (Fig 1C), or citizenship status (Fig 1D).

As we observed no significant correlation between admissions committee scores and time to degree, we next examined whether 1) students in each group graduate at different rates (i.e., changes in slope), and/or 2), if the threshold (y-intercept) at which these students graduate is different. We used a method previously described [24]. Briefly, a difference in slope as a measurement of the rate of graduation would suggest that students in different groups graduate at different rates. A difference in threshold between two groups indicates that the starting point for the graduation rate is different. For example, it is possible for students in two different groups to graduate at the same rate (slope) but have vastly different thresholds at which they graduate (*y*-intercept) In our analysis, we did not observe a significant difference in graduation rate or threshold based on gender, minority status, or citizenship status (Table 1) suggesting that, on their own, admissions committee scores cannot reliably predict graduate outcomes for PhD students.

**Table 1:** Summary of statistical analysis regarding admissions scores and time to degree.

Summary of Statistical Analyses of Pre-Intervention Data (2007–2012)			
	Correlation between Admissions Score and TTD	Linear regression	
	p-value (Pearson correlation coefficient)	Difference in slope (rate of graduation) p-value	Difference in elevation (threshold) p-value
All students	0.3373 (-0.05634)	NA	NA
Males	0.7046 (-0.03438)	0.7981	0.5254
Females	0.341 (-0.07392)		
Well-represented	0.7123 (-0.03100)	0.2661	0.4902
Under-represented minority	0.2398 (-0.1854)		
Domestic	0.4891 (-0.05103)	0.9657	0.883
International	0.4908 (-0.06766)		

Table 1 summarizes the statistical analyses that were conducted on each data set regarding admissions scores and time to degree. None of the data that was analyzed showed statistically significant correlations or differences in slope or elevation regardless of gender (Fig 1B), race and ethnicity (Fig 1C), or citizenship (Fig 1D).

### Undergraduate GPA can predict time to degree

A major component of the application file is a student’s academic transcript as it provides information regarding the applicant’s previous academic performance. Like GRE scores, the GPA can appear to be a normalized quantitative measure through which applicants can be compared and thus is often used as a quantitative cutoff. While the GPA can provide information such as which courses the student has taken and the grade that the student received, it fails to include important factors such as classroom size, quality of education, access to resources, and socioeconomic status among others. Furthermore, there are conflicting reports about its reliability as a predictive measure for graduate outcomes [31, 34–36].

**Fig 2:**
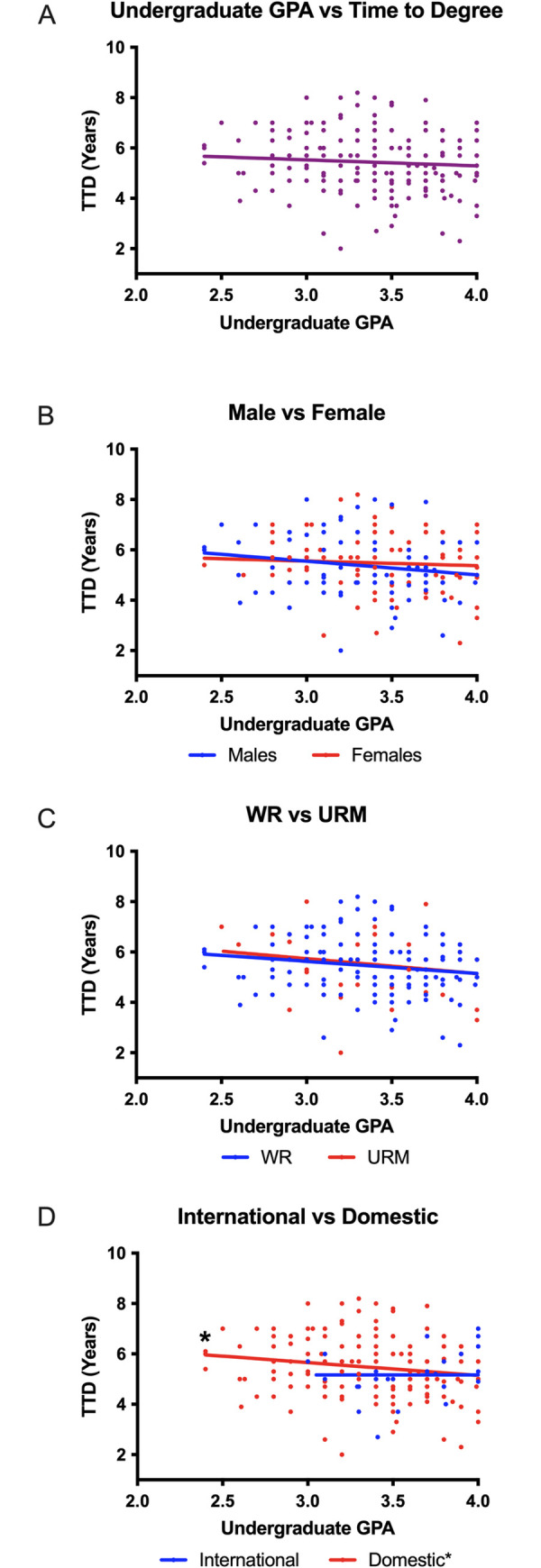
Undergraduate GPA is significantly correlated to the time that it takes to graduate with a PhD for domestic students when compared to international students. Time to degree is plotted as a function of undergraduate GPA (A). Further analysis compared undergraduate GPA and time to degree based on gender (B), representation (C), and citizenship (D).

**Fig 3:**
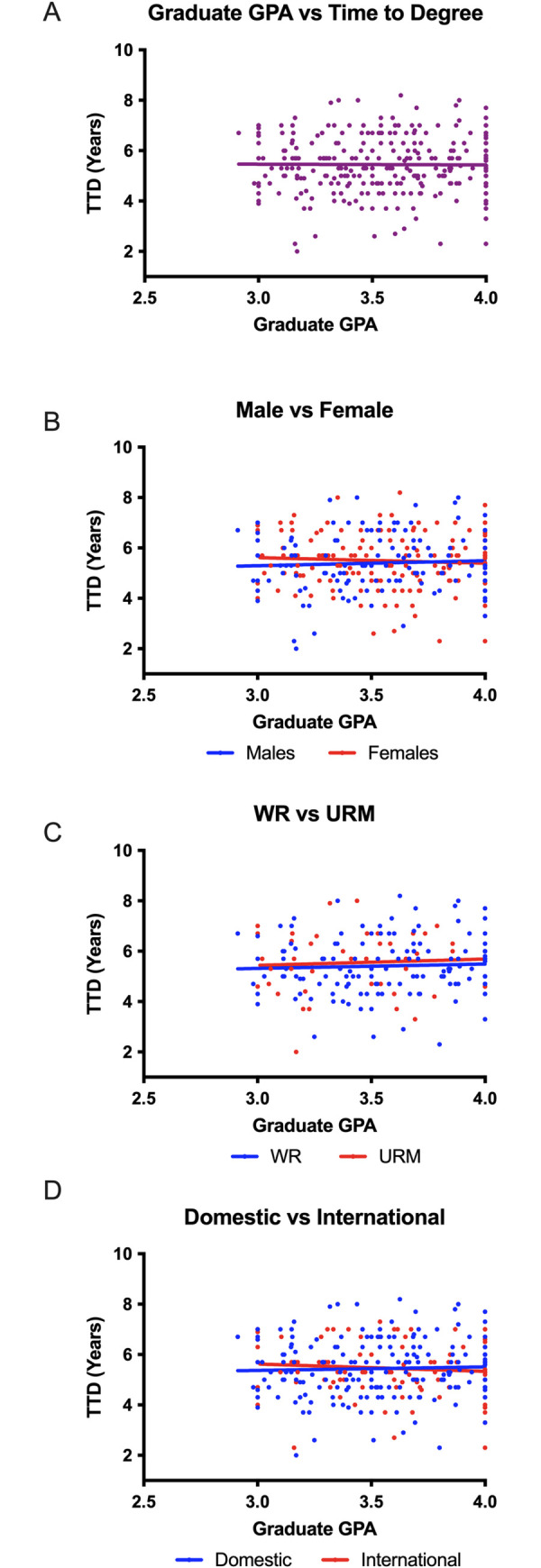
Graduate level GPA is not significantly correlated to the time it takes students to graduate with a PhD. Time to degree is plotted on a scatterplot as a function of graduate GPA (A). The data was further divided on a basis of gender (B), representation (C), and citizenship (D).

While undergraduate GPA does not vary significantly between the top and bottom performers in graduate programs [34], when compared to other predictive measures such as standardized testing, GPA is one of the most reliable predictors that is used in the admissions process [31, 35, 36]. In the absence of statistically significant correlations between admissions committee scores and TTD (Fig 1 and Table 1), we examined whether GPA could predict outcomes for PhD students in our program. We assessed whether there were any trends between undergraduate GPA (x-axis) and TTD (y-axis) (Fig 2). We observed a negative trend between undergraduate GPA and time to degree for all students (Fig 2A) suggesting that the higher a student’s undergraduate GPA, the less time it would take for that student to graduate with a PhD. Interestingly, when we analyzed the data by gender, race, ethnicity, and citizenship status, significance was only observed for domestic students (Table 2 and Fig 2D). Similar to previous findings [31, 35, 36], we observed a correlation between GPA and TTD, however, our analysis suggests that one size does not fit all when using GPA as a predictor of PhD program success. We also observed no significant correlations between graduate GPA and TTD outcomes (Fig 3 and Table 3).

**Table 2:** Summary of statistical analysis regarding undergraduate GPA and time to degree.

Summary of Statistical Analyses of Pre-Intervention Data (2007–2012)			
	Correlation between UG GPA and TTD	Linear regression	
	p-value (Pearson correlation coefficient)	Difference in slope (rate of graduation)	Difference in elevation (threshold)
		p-value	p-value
All students	0.0937 (-0.1197)	NA	NA
Males	0.1203 (-0.1740)	0.3155	0.4084
Females	0.2242 (-0.1137)		
Well-represented	0.0682 (-0.1593)	0.8393	0.755
Under-represented minority	0.2741 (-0.1795)		
Domestic	0.0307* (-0.005467)	0.0903	0.4152
International	0.9789 (-0.1653)		

Table 2 outlines the statistical analyses that were conducted on each data set regarding undergraduate GPA and time to degree. The only statistically significant correlation that was observed was for domestic students with a p value of 0.0307. No statistically significant differences in slope or elevation were observed in any categories.

*p ≤ 0.05

**Table 3:** Summary of statistical analysis regarding graduate GPA and time to degree.

Summary of Statistical Analyses of Pre-Intervention Data (2007–2012)			
	Correlation between GSBS GPA and TTD	Linear regression	
	p-value (Pearson correlation coefficient)	Difference in slope (rate of graduation)	Difference in elevation (threshold)
		p-value	p-value
All students	0.8923 (-0.007959)	NA	NA
Males	0.5565 (0.05331)	0.3182	0.5179
Females	0.3987 (-0.06554)		
Well-represented	0.5643 (0.04843)	0.9156	0.4588
Under-represented minority	0.7297 (0.05494		
Domestic	0.6104 (0.03760)	0.3135	0.9846
International	0.3617 (-0.08948)		

Table 3 describes the findings from statistical analyses that were conducted on the data regarding graduate GPA and time to degree. None of the data analyzed showed any statistically significant correlations or differences in elevation or slope.

To further understand the relationship between GPA and graduation rate, we conducted linear regression analysis to determine whether there was a difference in rate of graduation (slope) or threshold (*y*-intercept) between each group. A difference in slopes would imply a difference in rate of graduation based on GPA while differences in elevations would suggest that the threshold (y-intercept) for graduation based on GPA is significantly different between groups. We observed no significant differences in graduation rate or threshold between any of the groups based on undergraduate GPA (Table 2) or graduate GPA (Table 3). Since there is a significant correlation between undergraduate GPA and time to degree for domestic students and no correlations between graduate GPA and time to degree, these data indicate that undergraduate GPA is a better predictor of TTD than graduate GPA.

### GRE scores predict time to degree for URMs and international students

**Fig 4:**
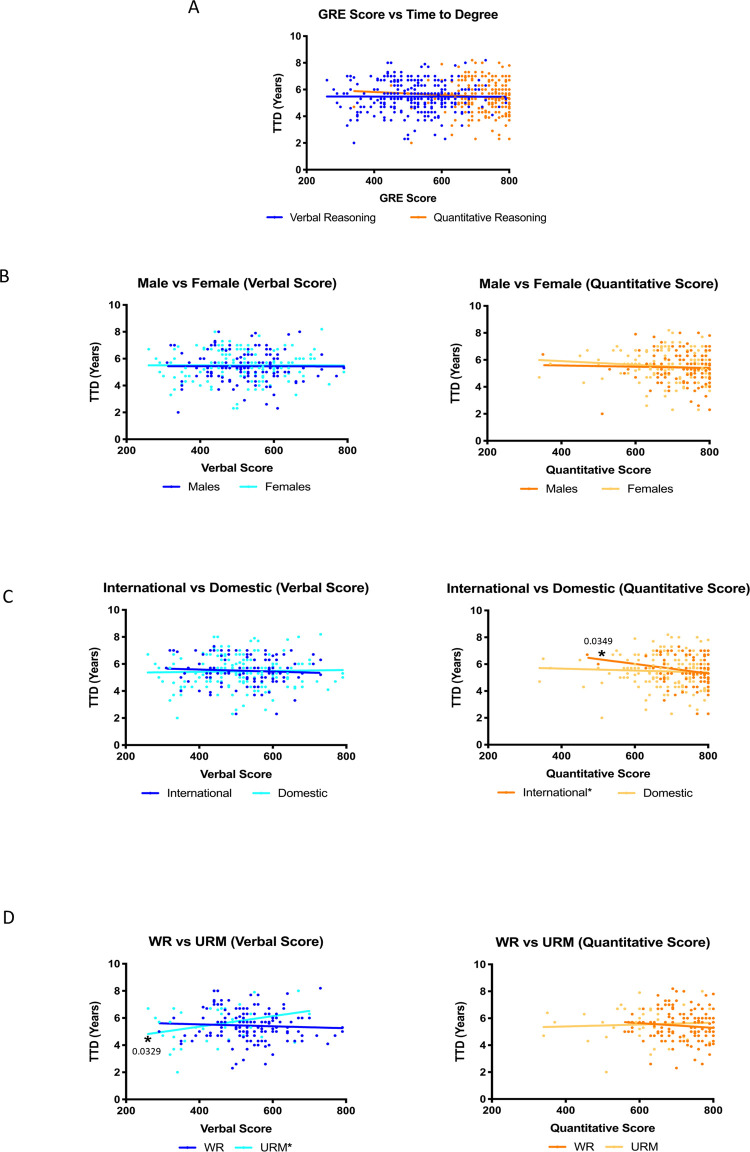
GRE scores are significantly correlated to the time it takes students to graduate with a PhD. Time to degree is plotted on a scatterplot as a function of GRE score (A). The data was further divided on a basis of gender (B), citizenship (C), and representation (D).

We next examined whether GRE scores could predict TTD for PhD students. We used a scatterplot analysis to analyze possible relationships between GRE score and either the verbal or quantitative section of the exam (x-axis) and TTD (y-axis) (Fig 4). While we did not observe significant correlations between GRE score and TTD for all students (Fig 4A), when data were analyzed based on race, ethnicity, and citizenship, we observed two significant correlations. First, there was a negative correlation between quantitative GRE score and TTD for international students. These data suggest that the higher an international student scores on the quantitative portion of the GRE, the less time it will take for that student to complete the program. Second, there was a positive correlation between the verbal GRE score and TTD for URMs. This correlation indicates that the higher a URM student scores on the verbal assessment of the GRE, the longer it will take for this student to earn their PhD.

We next analyzed the relationship between GRE scores and graduation rate via linear regression analysis to determine whether there was a difference in rate of graduation (slope) or threshold (y-intercept). Like our analysis of GPA and TTD, a difference in slopes would imply a difference in rate of graduation based on race, ethnicity, or citizenship status. A difference in elevations would suggest that the threshold (y-intercept) for graduation based on race, ethnicity or citizenship status is significantly different between groups. We observed a significant difference in slope between well-represented students and URMs based on GRE verbal scores (Table 4). This difference further supports the correlation that we found in URMs, as the higher a URM student’s verbal GRE score, the more time it will take for the student to complete the program.

**Table 4:** Summary of statistical analysis regarding GRE scores and time to degree.

Summary of Statistical Analyses of Pre-Intervention Data (2007–2012) (Scores)							
		Correlation between GRE and TTD		Linear regression			
	Number	Quantitative p-value (Pearson correlation coefficient)	Verbal p-value (Pearson correlation coefficient)	Difference in slope (rate of graduation)		Difference in elevation (threshold)	
				Quantitative p-value	Verbal p-value	Quantitative p-value	Verbal p-value
All students	286	0.1542 (-0.08447)	0.9529 (-0.00350)	0.2858		0.5028	
Males	120	0.7613 (-0.02802)	0.9746 (-0.00294)	0.59	0.9929	0.8425	0.6251
Females	166	0.1371 (-0.1159)	0.9753 (-0.00241)				
Well-represented	141	0.234 (-0.1009)	0.4842 (-0.05939)	0.2711	0.0197*	0.8719	NA
Under-represented minority	42	0.6997 (0.06131)	0.0329* (0.3299)				
Domestic	183	0.4573 (-0.05529)	0.6823 (0.03046)	0.162	0.4198	0.4548	0.7998
International	103	0.0349* (-0.2081)	0.4623 (-0.07323)				

Table 4 outlines the statistical analyses that were conducted on the data regarding GRE scores and time to degree. Statistically significant correlations were observed for international student Quantitative GRE scores and TTD with a p-value of 0.0349, and URM student Verbal GRE scores and TTD with a p-value of 0.0329. Data analyzed showed a statistically significant difference in slope (rate of graduation) based on representation with a p-value of 0.0197.

*p ≤ 0.05

### GRE percentiles predict time to degree for well-represented students

While there are multiple published studies that analyze the predictive validity of GRE scores [28–30, 33], there are none that analyze the predictive validity of GRE percentiles. There are two limitations of using GRE scores: 1) The GRE scoring system was changed in 2011 from a range of 200–800 points per section to a range of 130–170 points per section and 2) GRE scores do not compare a student’s GRE performance to other students that took the GRE around the same time. We wanted to analyze the predictive validity of GRE percentiles because they are a more normalized measure of GRE performance than GRE scores and they compare the performance of each student to other students within the same reference group. In fact, percentile ranks are adjusted to normalize GRE scores over time [1].

**Fig 5:**
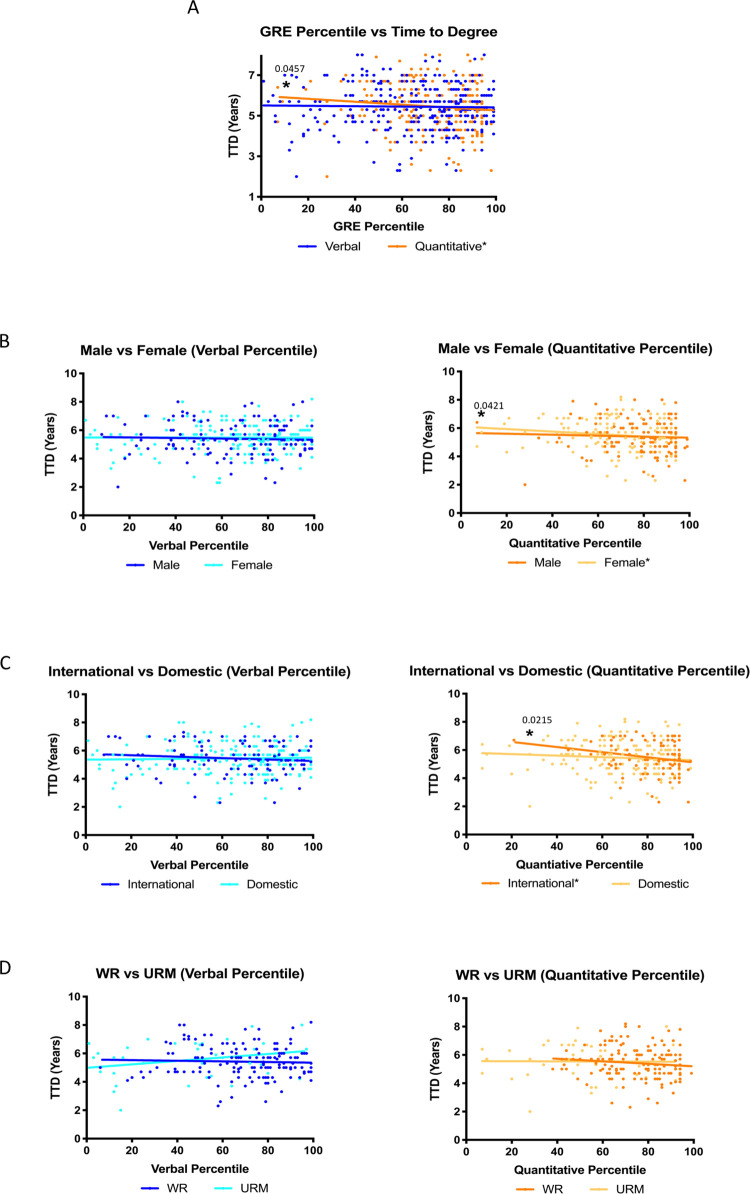
Quantitative GRE percentiles are significantly correlated to the time it takes students to graduate with a PhD. Time to degree is plotted on a scatterplot as a function of GRE percentile (A). The data was further divided on a basis of gender (B), citizenship (C), and representation (D).

We analyzed whether GRE percentiles are correlated with TTD by comparing GRE percentiles (x-axis) and TTD (y-axis) (Fig 5). Unlike what we observed with GRE scores, there was a negative correlation between GRE percentile on the quantitative section of the exam and TTD for all students (Fig 5A). Furthermore, when we analyzed the data based on gender, race, ethnicity, and citizenship status, we observed a negative correlation in female students (Fig 5B) and international students (Fig 5C) with GRE quantitative percentiles. These correlations suggest that: 1) there are differences between GRE scores and percentiles and 2) the higher a student’s GRE quantitative percentile, the less time it will take for the student to graduate with a PhD. There were no significant differences in graduation rate or threshold based on gender, race, ethnicity, or citizenship (Table 5).

**Table 5:** Summary of statistical analysis regarding GRE percentiles and time to degree.

Summary of Statistical Analyses of Pre-Intervention Data (2007–2012) (Percentile)							
		Correlation between GRE and TTD		Linear regression			
	Number	Quantitative p-value (Pearson correlation coefficient)	Verbal p-value (Pearson correlation coefficient)	Difference in slope (rate of graduation)		Difference in elevation (threshold)	
				Quantitative p-value	Verbal p-value	Quantitative p-value	Verbal p-value
All students	292	0.0457* (-0.117)	0.726 (-0.02059)	0.1681		0.737	
Males	124	0.5948 (-0.04822)	0.636 (-0.04292)	0.485	0.7314	0.8356	0.5353
Females	168	0.0421* (-0.157)	0.9773 (-0.002214)				
Well-represented	144	0.1679 (-0.1155)	0.6321 (-0.04023)	0.42	0.0766	0.9708	0.4023
Under-represented minority	42	0.9612 (-0.007736)	0.0936 (0.2621)				
Domestic	186	0.2212 (-0.09012)	0.6922 (0.02922)	0.1696	0.2568	0.4754	0.9922
International	106	0.0215* (-0.2231)	0.2434 (-0.1143)				

Table 5 outlines the statistical analyses that were conducted on the data regarding GRE percentiles and time to degree. Statistically significant correlations were observed for all students Quantitative GRE percentiles and TTD with a p-value of 0.0457. Statistically significant correlations were also observed for Quantitative GRE percentiles and TTD based on gender and citizenship with p-values of 0.0421 and 0.0215 respectively. Data analyzed does not show a statistically significant difference in slope or threshold for any group.

*p ≤ 0.05

### GRE scores or GPA alone cannot accurately predict candidacy exam success while GRE percentiles can predict candidacy exam outcomes

Previous work has shown that neither GRE scores nor undergraduate GPA predict PhD candidacy examination results [28]. Moneta-Koehler *et al*. analyzed relationships between GRE scores and undergraduate GPA with PhD candidacy examination success using a linear probability model. This method uses a binary categorical variable on the y-axis and assigns a value of either one or zero to the potential outcomes. In this work, a one represents a passing value while a zero represents a failed candidacy exam [28, Table A]. The data was then arranged in a linear regression model and the probability of a student passing the candidacy exam was calculated based on GRE score or GPA. While the authors observed no relationship between GRE score or GPA and candidacy exam results, there are two major limitations of this study. First, the outcome is binary which means that there can be only two results (either the student passes or fails the candidacy exam), however, the linear probability model provides a range of probabilities. Some of these probabilities can fall outside of the accepted span of results from zero to one leaving portions of the data uninterpretable. Second, linear probability models are prone to heteroskedasticity or an uneven variation of data which causes standard error estimates of the model to be too large to yield accurate results.

**Fig 6:**
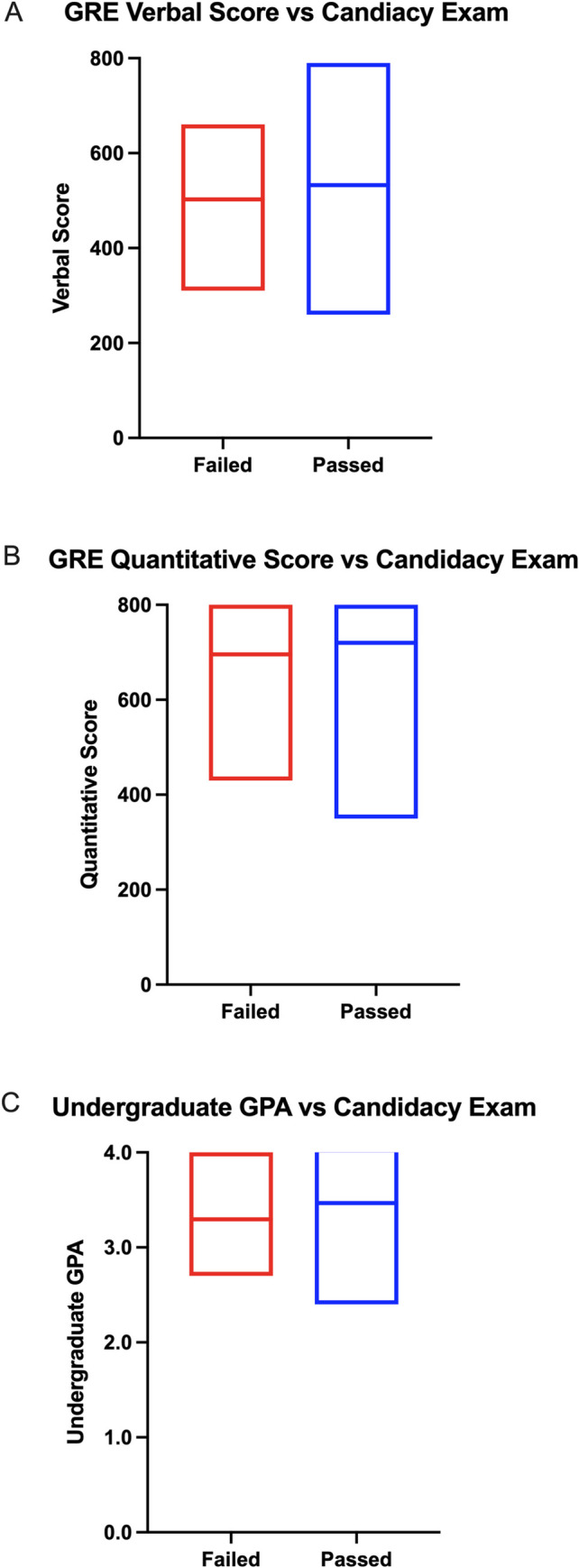
There is no statistically significant variation in mean GRE scores or undergraduate GPA between students who passed or failed the PhD candidacy examination. Candidacy exam outcome (x-axis) is compared to GRE verbal score (A), GRE quantitative score (B), and undergraduate GPA (C) (y-axis).

To address this question in our data set, we analyzed whether GRE score or GPA could predict candidacy exam outcomes using a Mann-Whitney test. The Mann-Whitney test detects significant differences in the mean values between two or more categories. While both a linear probability model and the Mann-Whitney test are valid ways to examine the predictive validity of measures such as the GRE, we believe that the Mann-Whitney test is less susceptible to abnormal data and is more likely to produce a valid and interpretable result. To visualize our data, we used a column analysis that compared the average GRE scores and undergraduate GPAs (y-axis) of students who either passed or failed the candidacy exam (x-axis) (Fig 6). If the average predictive metric was significantly different between students who passed or failed the candidacy exam, then there would be evidence to suggest that there is validity in using that metric as a predictor of candidacy exam success. Like Moneta-Koehler *et al*., we did not observe a significant relationship between undergraduate GPA or GRE score and candidacy exam outcomes (Table 6).

**Table 6:** Summary of statistical analysis regarding GPA and GRE scores and candidacy exam passage or failure.

Summary of Statistical Analyses of Pre-Intervention Data (2007–2012)	
	Difference in Mean Between Passed and Failed
	p-value
GRE Verbal Scores	0.196
GRE Quantitative Scores	0.1619
Undergraduate GPA	0.1142

Table 6 displays the p-value from the Mann-Whitney test regarding the difference in mean GRE scores or GPA between students who passed or failed the candidacy exam. None of the data analyzed showed a significant difference in average GRE score or GPA between candidacy exam results.

**Fig 7:**
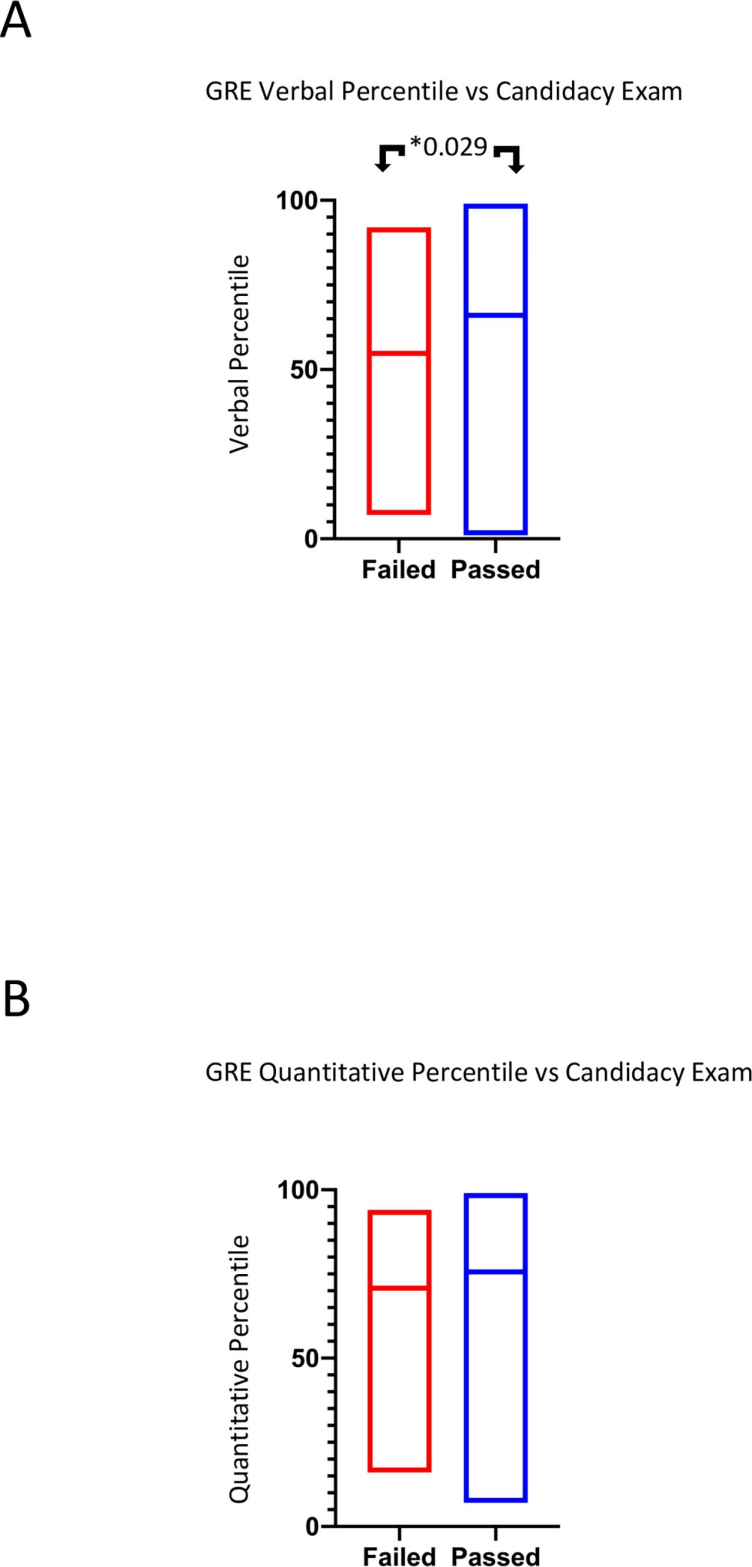
There is a statistically significant variation in mean GRE percentile between students who passed or failed the PhD candidacy examination. Candidacy exam outcome (x-axis) is compared to GRE verbal percentile (A), and GRE quantitative percentile (B) (y-axis).

Lastly, since we have observed differences in the relationship between GRE percentiles and GRE scores and their ability to predict TTD, we assessed whether GRE percentiles can predict candidacy exam outcomes. To this end, we used a Mann-Whitney test and column analysis that compared GRE percentile (y-axis) to candidacy exam result (x-axis) (Fig 7). Contrary to our findings comparing GRE scores and GPA with candidacy exam results, we observed a significant difference in average verbal GRE percentile between students who passed or failed the candidacy exam (Fig 7A and Table 7). These data suggest that, while GRE scores or GPA alone cannot predict candidacy exam results, GRE verbal percentiles can predict candidacy exam outcomes.

**Table 7:** Summary of statistical analysis regarding GRE percentiles and candidacy exam passage or failure.

Summary of Statistical Analyses of Pre-Intervention Data (2007–2012)	
	Difference in Mean Between Passed and Failed
	p-value
GRE Verbal Percentiles	0.0299*
GRE Quantitative Percentiles	0.2345

Table 7 displays the p-value from the Mann-Whitney test regarding the difference in mean GRE percentiles between students who passed or failed the candidacy exam. Data analyzed showed a significant difference in average Verbal GRE percentile between candidacy exam results with a p-value of 0.0299.

*p ≤ 0.05

## Discussion

Graduate programs in the biomedical sciences can shape the STEM workforce by admitting students who will develop a range of skills that will contribute to the future of the field. It is therefore important that admissions committees have a thorough understanding of the materials in an application packet and how they can be used to predict which applicants will be most likely to successfully complete their programs of study. While multiple authors have analyzed the predictive validity of the undergraduate grade point average (GPA) and the GRE [28–30, 32, 34–36], to our knowledge this is the first complete analysis to include GRE percentiles. We observed that GRE ***percentiles*** could predict candidacy exam results (Fig 7 and Table 7) but GRE ***scores*** did not (Fig 6 and Table 6). Additionally, we observed significant correlations between GRE *percentiles* and time to degree (TTD) in some cases (Fig 5 and Table 6). On the contrary, GRE *scores* had a more nuanced relationship with graduate outcomes (Fig 4 and Table 4). While negative correlations between GRE *percentiles* and TTD existed for many groups, GRE *scores* were only negatively correlated with TTD for international students. Furthermore, a positive correlation existed between GRE *scores* and TTD for URMs, suggesting that the higher a URM’s GRE score, the longer it will take for a student to complete the PhD program. One possible explanation is that URMs in STEM programs are less likely to pursue faculty positions at academic research institutions [21]. As a result, it is possible that URMs are more likely to remain in graduate programs while considering an alternative career. Finally, there may be bias regarding standards of graduation for different racial and ethnic groups among individual faculty members. Thus, we conclude that additional studies are needed to examine the reasons why URM doctoral students with high GRE verbal scores have extended times to degree completion.

In addition to our findings regarding the GRE, we observed correlations between undergraduate GPA and TTD (Fig 2 and Table 2). Although significance was not observed for all students, the presence of an inverse correlation between TTD and GPA for domestic students suggests that the undergraduate GPA does have predictive validity in all cases. It is possible that, when used as part of a holistic review, the predictive capability of the undergraduate GPA would increase for all applicants regardless of gender, race, ethnicity, or citizenship status. While we did not observe significant correlations between graduate GPA and TTD for any of the groups analyzed (Fig 3 and Table 3), we have identified three possible explanations for this discrepancy. First, graduate students usually take fewer courses at one time than undergraduate students. This means that graduate students are more likely to devote significant time to understanding focused subject matter compared to an undergraduate curriculum. Second, grades earned in research-oriented PhD Programs are typically de-emphasized compared to undergraduate programs. The resulting restricted range of grades could skew graduate GPAs and make them a less reliable measure of a student’s understanding than undergraduate GPAs. In addition, undergraduate faculty typically assign grades based on an understanding of fundamental concepts, while graduate faculty often assess problem solving skills. Third, often graduate courses are graded in a pass/fail format which could also alter the graduate GPA and minimize its predictive validity.

Our findings are unique because we have observed differences in the predictive ability of GRE *scores* and *percentiles*. Additionally, we have observed statistically significant data that suggests that both undergraduate GPA and GRE *percentiles* have predictive validity with respect to certain populations and certain outcomes. Many investigators that have analyzed the predictive validity of the GRE describe negative data or an absence of significant relationships between GRE scores and certain measures of graduate school success such as TTD or candidacy examination results. However, our findings indicate that when considered appropriately as a tool of holistic review admissions, quantitative metrics can be a valid predictor of graduate school success. As we previously demonstrated [24], a whole-file review that uses these quantitative metrics can both mitigate the reported bias of these measures and harness their predictive validity. Thus, we propose the following recommendations for graduate biomedical sciences admissions committees and programs:

1. *The use of a nuanced*, *holistic review that considers both qualitative and quantitative data*. While the GRE is a standardized examination, there is a level of careful consideration of scores and percentiles necessary to fully interpreting its results. First, while percentiles on the quantitative section of the GRE are significantly correlated to TTD for all students, they could not predict candidacy examination results. Instead, percentiles on the verbal section of the GRE are predictive of candidacy exam results but not TTD. The difference in predictive capability between the verbal and quantitative sections of the GRE suggest that admissions committees should take GRE performance on both sections into account when evaluating applicants holistically. Second, quantitative metrics can be predictive of graduate school success in non-obvious ways. While GPA is correlated with TTD for domestic students, GRE quantitative percentiles are predictive of TTD for female and international students. As the predictive validity of these metrics varies by demographic group, admissions committees should be cautious when using them to make predictions regarding the likelihood of applicants to complete the program.
2. *The use of GRE percentiles instead of GRE scores*. We have observed that GRE *scores* and *percentiles* are differently correlated with graduate school outcomes depending on the demographic of the population being analyzed. While GRE scores on the quantitative section of the exam are correlated with TTD for international students, percentiles on the quantitative section are predictive of TTD for all applicants. Additionally, percentiles on the verbal section of the GRE are predictive of candidacy examination results. Thus, when using quantitative metrics in a holistic review, GRE percentiles are significantly better predictors of graduate school success than GRE scores.
3. *Targeted curriculum supporting graduate student development of critical thinking and analytical skills*. By its very nature, obtaining the PhD is an exercise in developing the ability to critically think and analyze complex problems. As one key transition phase in STEM careers, the progression from pre-candidacy to post-candidacy is a common measure of success in STEM PhD programs. We have observed that percentiles on the verbal section of the GRE, which examines the ability of an applicant to comprehend complex literature and form conclusions based on the information provided, are predictive of candidacy exam outcomes. As such, implementation of programing and targeted curriculum designed to aid in the development of a PhD student’s critical and analytical thinking skills pre-candidacy can increase the success of these students as measured by candidacy exam outcomes.

## Limitations of this study

There are limitations to the data and analysis presented in this study. While time to degree and candidacy examination results are two important measures of graduate school progress in any program, there are other output variables that we did not measure such as publication count or performance in graduate level classes. Our dataset was also limited as it only consisted of students that had enrolled in and completed the PhD program and does not include datapoints for all metrics considered in the holistic review process (e.g. prior publication, postbac program attendance, etc.) In addition, this was a highly contextualized study that only analyzed students at The University of Texas MD Anderson Cancer Center UTHealth Graduate School of Biomedical Sciences. Finally, the size of the sample is relatively small compared to the national population of doctoral students. This sample size could limit the predictive ability of certain statistical analyses and caution should be exercised when applying our recommendations to larger groups of PhD students.

## Supporting information

S1 Fig Data analysis flow chart. A flow chart detailing criteria used to determine participant record inclusion or exclusion in data analysis.
(PDF)
