# Supplementary material for: The impact of holistic review on correlations between doctoral student outcomes, and GPA and GRE scores in the biomedical sciences
Source: PLoS One. 2022 Dec 16;17(12):e0279258. doi: 10.1371/journal.pone.0279258 (PMC9757574; doi:10.1371/journal.pone.0279258)
Supplement: S1 Fig — A flow chart detailing criteria used to determine participant record inclusion or exclusion in data analysis. (PDF) [file pone.0279258.s001.pdf]

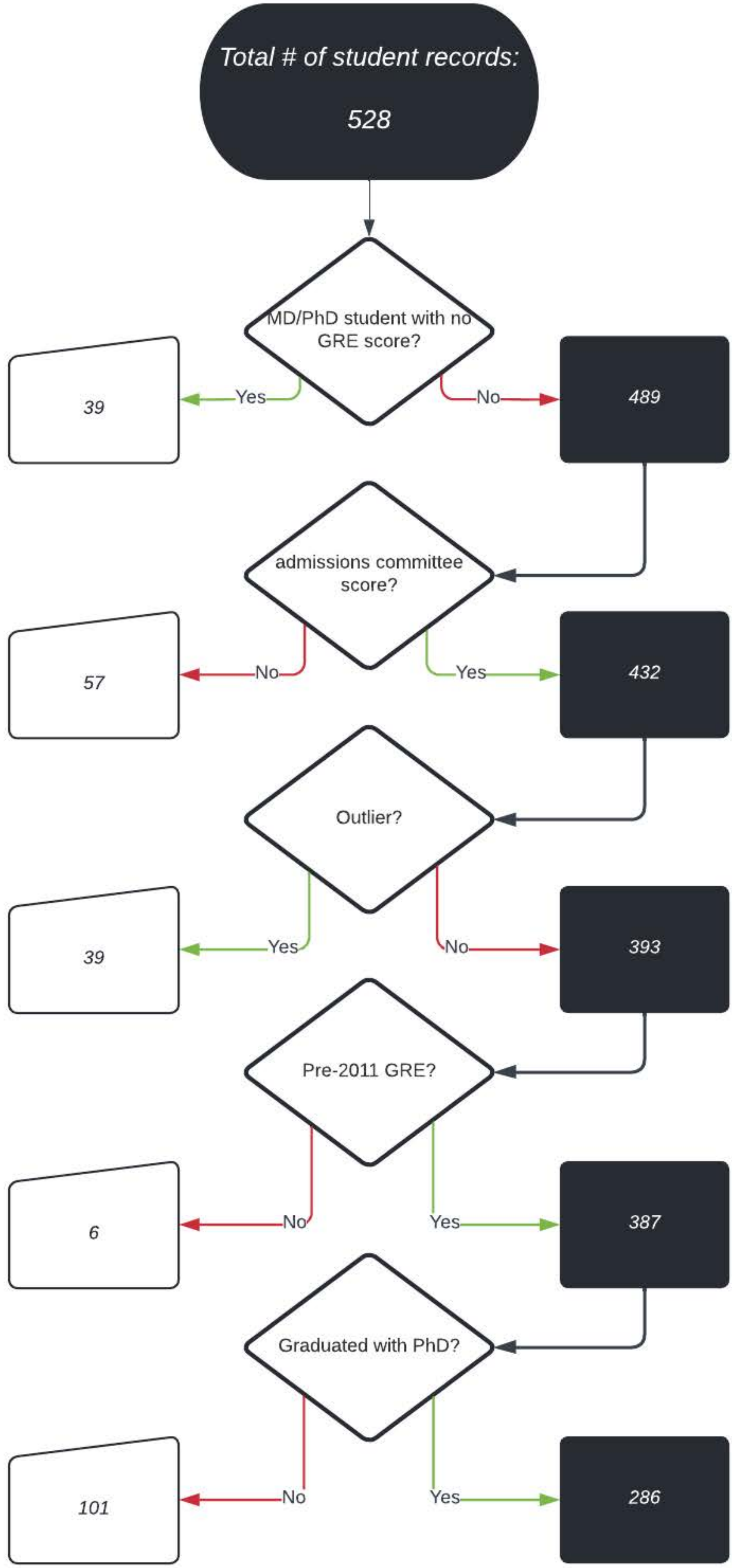

Total # of student records:
528
MD/PhD student with no GRE score?
Yes
39
No
489
admissions committee score?
No
57
Yes
432
Outlier?
Yes
39
No
393
Pre-2011 GRE?
No
6
Yes
387
Graduated with PhD?
No
101
Yes
286
